# Efficient Production of High Specific Activity Thulium-167 at Paul Scherrer Institute and CERN-MEDICIS

**DOI:** 10.3389/fmed.2021.712374

**Published:** 2021-10-12

**Authors:** Reinhard Heinke, Eric Chevallay, Katerina Chrysalidis, Thomas E. Cocolios, Charlotte Duchemin, Valentin N. Fedosseev, Sophie Hurier, Laura Lambert, Benji Leenders, Bruce A. Marsh, Nicholas P. van der Meulen, Peter Sprung, Thierry Stora, Marianna Tosato, Shane G. Wilkins, Hui Zhang, Zeynep Talip

**Affiliations:** ^1^Institute for Nuclear and Radiation Physics, KU Leuven, Leuven, Belgium; ^2^European Organization for Nuclear Research CERN, Geneva, Switzerland; ^3^Belgian Nuclear Research Centre SCK CEN, Mol, Belgium; ^4^Department of Electromechanical, Systems and Metal Engineering, Ghent University, Ghent, Belgium; ^5^Laboratory of Radiochemistry, Paul Scherrer Institute, Villigen, Switzerland; ^6^Center for Radiopharmaceutical Sciences ETH-PSI-USZ, Paul Scherrer Institute, Villigen, Switzerland; ^7^Analytic Radioactive Materials, Paul Scherrer Institute, Villigen, Switzerland; ^8^Division Large Research Facilities, Paul Scherrer Institute, Villigen, Switzerland

**Keywords:** medical radionuclides, thulium-167, mass separation, laser resonance ionization, MEDICIS, Auger electrons

## Abstract

Thulium-167 is a promising radionuclide for nuclear medicine applications with potential use for both diagnosis and therapy (“theragnostics”) in disseminated tumor cells and small metastases, due to suitable gamma-line as well as conversion/Auger electron energies. However, adequate delivery methods are yet to be developed and accompanying radiobiological effects to be investigated, demanding the availability of ^167^Tm in appropriate activities and quality. We report herein on the production of radionuclidically pure ^167^Tm from proton-irradiated natural erbium oxide targets at a cyclotron and subsequent ion beam mass separation at the CERN-MEDICIS facility, with a particular focus on the process efficiency. Development of the mass separation process with studies on stable ^169^Tm yielded 65 and 60% for pure and erbium-excess samples. An enhancement factor of thulium ion beam over that of erbium of up to several 10^4^ was shown by utilizing laser resonance ionization and exploiting differences in their vapor pressures. Three ^167^Tm samples produced at the IP2 irradiation station, receiving 22.8 MeV protons from Injector II at Paul Scherrer Institute (PSI), were mass separated with collected radionuclide efficiencies between 11 and 20%. Ion beam sputtering from the collection foils was identified as a limiting factor. *In-situ* gamma-measurements showed that up to 45% separation efficiency could be fully collected if these limits are overcome. Comparative analyses show possible neighboring mass suppression factors of more than 1,000, and overall ^167^Tm/Er purity increase in the same range. Both the actual achieved collection and separation efficiencies present the highest values for the mass separation of external radionuclide sources at MEDICIS to date.

## 1. Introduction

Auger electrons (AEs) can be highly radiotoxic when they decay in the vicinity of DNA in the cell nucleus (1, 2), which makes them attractive for radiotherapy. Moreover, recent studies have shown that, even without nuclear localization, AEs can also kill targeted cancer cells by damaging the cell membrane, or non-targeted cells by a local cross-dose effect or a bystander effect (3). Nevertheless, to date, clinical research has been very limited and there is still much to learn about the molecular and cellular radiobiological effects of AEs. Most of the AE-emitting radionuclides also emit conversion electrons with higher energies compared to AEs, resulting in a longer-range effect up to several micrometers (4, 5) and less dense ionization.

Many research groups worldwide are focusing on the development of new targeting agents, however, the radionuclides have critical importance for the success of nuclear medicine applications. Previously, it was demonstrated that the decay of ^125^I in mammalian cell DNA leads to more than one double-strand break per decay (6). Examples of some of the interesting AE emitting radionuclides include ^99m^Tc (7), ^111^In (8), ^123^I (9, 10), ^125^I (11), ^201^Tl, ^119^Sb (12), ^67^Ga (13), ^191^Pt (14), ^193m^Pt, and ^195m^Pt. Due to the different chemical and pharmacokinetic properties, it is not possible to perform direct preclinical comparison studies with the radionuclides listed above except I and Pt radionuclides. Up to date, ^125^I is the most studied AE-emitter (15). Its long half-life (59.4 d) makes it less practical for the clinical applications. Moreover, low radioiodination efficiency (40–60%) precludes the kit formulation for the radiopharmaceutical preparation with ^123^I and ^125^I. Pt isotopes were shown to be highly radiotoxic due to the high emission rates (due to the excess of 30 Auger electrons per decay on average) (5). Nevertheless, low specific activity and no satisfactory radiolabeling strategies obstruct their further investigation.

Thanks to their similar chemical characteristics, such as analogous coordination chemistry, radiolanthanides have great potential to perform basic preclinical comparison studies to improve the understanding of AE therapy. ^167^Tm (t_1/2_ = 9.25 d) is a potential radionuclide both for medical diagnostics and therapy, due to its gamma emission (*E*_γ_ = 207.8 keV, *I* = 42%) for single-photon emission computed tomography (SPECT), and conversion/Auger electron emission (*E* = 5–100 keV, *I* = 120%) (16). Dosimetry calculations showed a high ratio of absorbed dose rate in the tumor with respect to the normal tissue (17, 18). In the past, ^167^Tm-citrate was used for tumor imaging (19) and comparative kinetic studies showed the advantage of ^167^Tm-citrate over simultaneously applied ^67^Ga-citrate in normal and tumor-bearing mice (19, 20). The very fast clearance from blood and good retention in tumor provided justifications for ^167^Tm to be more appropriate than ^67^Ga. However, suitable targeting methods are still to be developed and accompanying radiobiological effects have to be investigated. This is only possible if this radionuclide becomes available in appropriate activities and quality.

^167^Tm can be produced via several production routes by charged particle induced reactions (20–24). However, radionuclidically and chemically pure ^167^Tm batches for extensive studies can only be provided via a combination of mass and chemical separation. Key parameters are the overall efficiency and process duration. Respective operation parameters have to be tailored to the specific peculiarities of the isotope itself, as well as its chemical environment determined by the production path. Systematic preparatory studies are needed for optimization, to avoid losses in each single step, and also due to time constraints imposed by ongoing radioactive decay.

Mass separation at <100 keV ion beam energy electromagnetic mass separators, including sample preparation and, especially, ion source design and laser ionization system developments, has been proven to work efficiently for lanthanides in recent studies. Efficiency values in the range of a few 10% and up to more than 50% were reported for terbium (25), dysprosium (26), holmium (27), ytterbium (28), and lutetium (29) under optimum conditions.

This work focuses on the mass separation process at CERN-MEDICIS with ^167^Tm produced by proton irradiation from natural erbium oxide at PSI. It describes both the preparatory work with stable thulium, as well as the first three collections of ^167^Tm, in detail. As such, it illustrates the first step for the provision of this isotope by the collaboration, but also serves as general description of introducing a new chemical element/target matrix combination in a mass separation facility.

In addition, a full characterization of the samples via gamma-ray spectrometry, ICP-OES and ICP-MS validated their quality grade, toward their use in pre-clinical studies in the near future.

## 2. Experimental Setup and Methods

### 2.1. Production of ^167^Tm at PSI

^nat^Er_2_O_3_ target discs, 6 mm in diameter, were prepared and irradiated at the PSI's IP2 irradiation station (30), using the 72 MeV proton beam from Injector II separated sector cyclotron. Experimental cross-section results of ^nat^Er_2_O_3_ were previously reported by Tarkanyi et al. (31). It was shown that maximum cross-section results were obtained between 18 and 22 MeV. In this range, the obtained ^167^Tm production cross-section results are the sum of the ^167^Er(p,n) and ^168^Er(p,2n) reactions. In the present study, a 2.4 mm niobium disc was used as a degrader to decrease the proton energy to 22.8 MeV. The beam current was set to 50 μA and several test irradiations were performed using 30 mg ^nat^Er_2_O_3_ targets for 8 h. After irradiation, high dose rates were obtained due to the high-energy gamma rays of the co-product ^166^Tm (t_1/2_ = 7.7 h). As a result of dose optimization for transport classification and handling, transports of the samples were performed 7 days after irradiation to await the complete decay of ^166^Tm. Three targets, 106.13 (1,021), 97.81 (929), and 154.37 (1,482) MBq ^167^Tm, respectively, at time of shipping, were transported to the CERN-MEDICIS facility for mass separation. Errors are given as 2-sigma interval.

### 2.2. Mass Separation at MEDICIS

**Figure 1:**
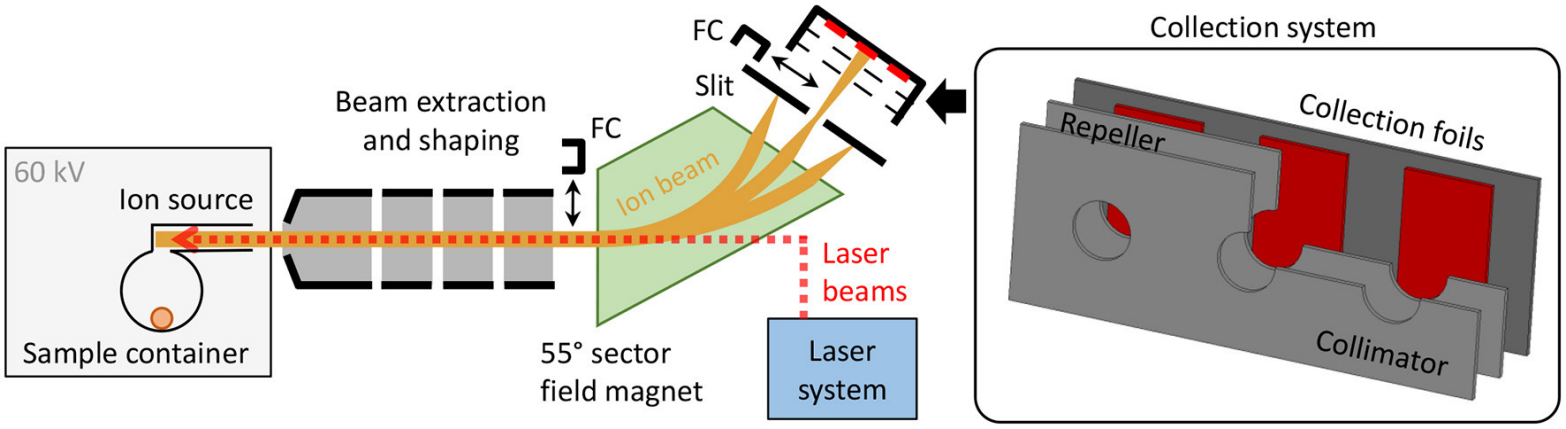
Schematic overview of the MEDICIS mass separator. The inset on the right-hand side schematically depicts a cut view of the actuatable collection system including the sample collection foils.

The CERN-MEDICIS (MEDical Isotopes Collected from ISolde) facility is dedicated to the production of non-conventional radionuclides for medical purposes (32–34). As schematically shown in Figure 1, it comprises a 60 kV ion beam dipole sector field magnet mass separator, that uses either targets that are irradiated in a dedicated irradiation station at the adjacent CERN-ISOLDE radioactive ion beam facility (33) by a 1.4 GeV proton beam or, alternatively, radiogenic samples produced and delivered by partner institutes. The radionuclide sample is evaporated and atomized in the ion source, and predominantly ionized by element-selective resonant laser radiation. The ion beam resulting from 60 kV extraction is separated according to the mass-over-charge ratio of its constituents. Faraday cups (FCs) can be inserted into the beam to monitor the beam current before and after separation. Desired ions are implanted into metallic foils situated in a holding system comprising collimator and electron repeller structures to allow for FC-like current monitoring. The sample collection system can be actuated during collection to switch between three different implantation foils. The samples are afterwards shipped to the end users for chemical purification and preclinical/clinical tests. A detailed technical overview of the MEDICIS separator, being the principal infrastructure of the presented work, is given in (35). The dedicated MEDICIS resonance ionization laser ion source system MELISSA (36, 37), resembling the solid-state laser system of ISOLDE RILIS (38), provides laser beams for element selective ionization by 10 kHz repetition rate wavelength-tunable Ti:sapphire lasers pumped by commercial frequency-doubled diode-pumped Nd:YAG lasers (Innolas Nanio).

For the case of thulium, laser resonance ionization inside a rhenium ion source was realized via a two-step two-resonance scheme developed at the TRILIS group at TRIUMF (39)^1^. A valence electron is excited from the atomic ground state into an intermediate state at 26889.12 cm^−1^ and, subsequently, into an auto-ionizing state at 51436.78 cm^−1^. The first step wavelength λ_1_ = 371.897 nm was provided with 500–700 mW, the second step wavelength λ_2_ = 407.371 nm with around 1.3 W at the output of the intra-cavity frequency-doubled Ti:sapphire lasers. In the initial setup and optimization phase, it was confirmed that both transitions were well-saturated, as expected from the reported saturation parameters of P_sat,1_ = 12 (1) mW and P_sat,2_ = 140 (21) mW (39). Additionally, the center wavelength of the resonances was confirmed.

### 2.3. Efficiency Assessment With ^169^Tm

A sample of stable ^169^Tm was produced by evaporating 10 μL of thulium standard solution (Alfa Aesar, 89889 Thulium, AAS standard solution, Specpure^Ⓡ^, Tm 1,000 μg/mL) on a rhenium coated tantalum cylinder (“boat”), which was afterwards placed in the center of the MEDICIS target container. This corresponds to an overall amount of 10 μg, 3.56 × 10^16^ atoms or 1,590 nAh integrated charge for singly-charged ions.

The mass separation process efficiency ϵ_sep_ of stable isotope separation processes can be determined by comparing the amount of collected atoms of choice *N*_coll_ at the end of the process to the amount of these atoms, which were initially present in the target container, *N*_sample_. Whereas, the latter is controlled by the amount of calibrated solution that is deposited, the former can be derived from the total collected charge *C*_coll_ of the ions at the collection point. This, in turn, can be calculated by time-integration of the ion current *I*_ion_ that is recorded after mass separation in a Faraday cup, where every ion contributes an elementary charge *e*.


(1) $$\begin{array}{l} \epsilon_{\text{sep}}=\frac{N_{\text{coll}}}{N_{\text{sample}}}=\frac{C_{\text{coll}}/e}{N_{\text{sample}}}=\frac{\int I_{\text{ion}}\text{d}t/e}{N_{\text{sample}}} \end{array}$$

For this type of measurement procedure a similar experimental setup on holmium, using neutron activation analysis for sample quantity confirmation (40), quantified the uncertainty in sample preparation to 4%, and the measured efficiencies from ion current were found to be consistent with the actual implanted isotope amount.

This efficiency value contains all single sub-processes that a sample atom has to undergo, i.e., diffusion and effusion out of possible matrix materials, transport into the ion source, transformation and persistence in its atomic form, ionization and non-neutralization, extraction, transport through the separator, and detection. A detailed breakdown and disentanglement of the individual contributions requires multiple dedicated experiments, yet, the overall value ϵ_total_ also gives a lower limit to each single one, assuming the others close to unity.

### 2.4. Efficiency Assessment With ^169^Tm in Erbium Oxide

In order to provide a more realistic test environment and to better evaluate the impact of the target material on the aforementioned individual factors involved in the efficiency, a second measurement was performed with the identical target container/ion source assembly. To an identically prepared 1,590 nAh sample of ^169^Tm, a huge excess quantity representative of an irradiated target of ^nat^Er_2_O_3_, used as material for ^167^Tm production, was added. Therefore, 100 mg of Er_2_O_3_ powder were dissolved in ethanol and the solution deposited and dried on the rhenium boat inside the sample container. This amount of Er_2_O_3_ corresponds to 87 mg of pure erbium, 3.1 × 10^20^ atoms or 14 mAh total charge for singly-charged ions. Thus, the ratio of ^169^Tm:^nat^Er was 1:8,800.

### 2.5. Efficiency Assessment With ^167^Tm From Proton-Irradiated Erbium Oxide

A series of mass separations on three ^167^Tm samples, produced as described in section 2.1, was performed at MEDICIS. These samples were weighed as 30–31 mg, thus, all containing around 9.4 × 10^19^ erbium atoms, respectively, corresponding to 4.2 × 10^6^ nAh for singly-charged ions. The ^167^Tm activity, after transport of the samples from PSI to MEDICIS and at the start of the actual collections, was 83.04 (799), 76.85 (730), and 122.94 (1,180) MBq, respectively. These activities correspond to contents of around 5 nAh, thus, the ^167^Tm:^nat^Er ratio in all samples was close to 1:10^6^.

Without any further treatment or dissolution, the irradiated erbium pellets were put in the rhenium boat and loaded into the target container. The same container/ion source unit as for the non-radioactive tests was used. The greatly reduced overall amount of thulium compared to the initial tests, in combination with the overwhelming background of surface-ionized erbium on this mass, did not allow for an initial optimization on a Faraday cup. Therefore, a ^169^Tm tracer amount about 30-fold more than the radiogenic ^167^Tm isotope was added in the same way as for the non-radioactive tests. This enabled optimization of the separator and laser operating parameters on mass 169 at still comparatively low container temperatures and thus low evaporation rates, conserving the radioactive sample. For the following actual ^167^Tm collection phase, the ion current was continuously monitored both on the implantation foil (0.25 mm thick gold foil coated with a 500 nm zinc layer) itself as well as on the collimator cover (diameter 10 mm) in front of it. Up to three implantation foils were used per run, while one each was used for internal tests and implanted with low activity.

### 2.6. Sample Characterization

Different techniques were applied to both the initial ^nat^Er_2_O_3_ targets and the mass-separated samples at MEDICIS and PSI, to characterize and quantify the production and separation process with respect to different quality parameters.

#### 2.6.1. Activity Measurements

At PSI, gamma-ray spectrometry measurements were performed using a high-purity germanium (HPGe) detector (Canberra, France). Full energy peak (FEP) efficiency calibration of the spectrometer was performed using a certified ^152^Eu point-like source [provided by Physikalisch-Technische Bundesanstalt (PTB), 1.49 × 10^5^ Bq, 01.01.2015]. The spectra were analyzed with Canberra's Genie 2000 software package. The samples were measured 7 days after irradiation, after mass separation and after dissolving the zinc layer, to determine the mass separation collection efficiencies and the remaining activities on the gold foils, respectively. Half-lives and characteristic gamma-lines of the Tm radionuclides used for calculations were as follows:

- ^167^Tm (t_1/2_: 9.25 d, 207.8 keV (42%) (16)
- ^168^Tm (t_1/2_: 93.1 d, 815.99 keV (50.95%) (41)
- ^165^Tm (t_1/2_: 30.06 h, 242.92 keV (35.5%) (42)

Deadtime of the measurements was <6%. The gamma-ray spectrometry measurements were performed with a 300 cm sample detector distance. Therefore, the geometry of the samples was considered as a point source.

At MEDICIS, the present ^167^Tm amount in the collection position was determined *in-situ* during the collection with a compact cadmium zinc telluride (CZT) gamma-ray spectrometry detector GR1 (Kromek, UK) fixed in front of the collection chamber. The device was FEP calibrated with certified sources (^60^Co, ^133^Ba, ^137^Cs, ^152^Eu) at a setup resembling the geometry beforehand (43). The count rate on the 207 keV gamma line of ^167^Tm was utilized. After collection, a dedicated measurement of each foil was performed by the CERN radiation protection service using a HPGe coaxial detector (Mirion Technologies). For absolute efficiency calibration, the ISOCS (*In Situ* Object Counting System) calibration software (44) was used, which allows to produce an accurate quantitative gamma assay of almost any sample type and size. After the measurements, the Genie 2000 software was used to analyze the acquired spectra and extract the corresponding activities. Corrections regarding counting statistics, detector dead time, systematic uncertainties of the peak area fitting, gamma emission probabilities and sample geometry model uncertainties were considered to determine the errors and limits of detection.

#### 2.6.2. Isotope Ratio Measurements

Three ^167^Tm-implanted Zn coated gold foils were introduced into a reaction vial and the Zn layer dissolved in 7 mL 6 M HNO_3_. The resultant solution was directly loaded onto a column containing N,N,N′,N′-tetra-n-octyldiglycolamide, non-branched resin (DGA, particle size 50–100 μm, TrisKem International, France; volume 0.08 mL), which is based on tetraoctyldiglycolamide as sorbent. The column was rinsed several times with 6.0 M HNO_3_ to remove the remaining Zn from the column. Thulium and erbium were, then, eluted using 5 mL 0.05 M HCl as it was reported in (45). These fractions were analyzed using ICP-MS and ICP-OES techniques.

A batch of ^nat^Er_2_O_3_, the test target and mass-separated samples from three collections were analyzed with the Nu Instruments Plasma 3 MultiCollector Inductively Coupled Plasma Mass Spectrometer, at PSI's Hot Laboratory, using an Elemental Scientific Apex HF nebulizing system and a self aspiring PFA nebulizer for sample introduction. This Plasma 3 is a sector-field mass spectrometer equipped with 16 Faraday detectors, three Daly detectors, and three secondary electron multipliers, ideal for measuring multiple ion beams simultaneously.

All samples and standards were introduced as acidic solutions in 0.28 M HNO_3_ and concentration-matched to yield similar ion beam intensities on mass 167. The online-measured ^176^Lu/^175^Lu ratio of admixed natural Lu was used to determine the magnitude of instrumental mass discrimination. Post-analyses and analyses of mixed solutions of natural Er and Lu were used to characterize the relation between the exponential mass discrimination factors (46) for Er and Lu [using isotope abundances from (47) and nuclide masses from (48)]. This relation, and the online-obtained exponential mass discrimination factors for Lu, subsequently allowed accurate mass discrimination corrections of Er isotope analyses irrespective of whether Er in a sample analysis exhibits natural isotope abundances. Ion beam intensities on stable-isotope masses of Yb and Dy were monitored for the mathematical correction, assuming natural isotope abundances (47) of isobaric Yb or Dy contributions to ion beams of Lu or Er masses of interest. These corrections were deemed insignificant for all reported results.

For the analyses of mass-separated samples, all ion beams were collected in Faraday detectors connected to amplifier systems having either 10^12^ Ω resistors in the feedback loop of their preamplifier (masses 166 and 168) or 10^11^ Ω resistors (all other masses). The fixed spacing of detectors on the Plasma 3 mass spectrometer did not allow for a simultaneous detection of ^176^Lu and ^175^Lu while measuring ion beams at masses 166 and 168 using the more sensitive 10^12^ Ω resistor-amplifiers. Therefore, a two-step dynamic analysis routine was chosen, in which the magnetic field was changed 20 times between two 15 s long measurement steps. Instrumental background signals were corrected using interspersed analyses of sample-and-standard-free 0.28 M HNO_3_. No isotope ratios involving Er masses 164 or 162 are reported because of Dy contributions to ion beams of these masses that were too high to correct for. Ion beam intensities on mass 167 were ca. 4 pA obtained from 100 ppb (element concentration) solutions of natural Er.

For analyses of the natural Er_2_O_3_ and its irradiated counterpart, all ion beams were detected simultaneously and, with the exception of mass 162, collected in Faraday detectors having either 10^12^ Ω resistors in the feedback loop of their preamplifier (^161^Dy) or 10^11^ Ω resistors (all other masses). The ion beam at mass 162 was detected using the more sensitive Daly ion counter given the low isotopic abundance of ^162^Er [0.139% (49)]. The approximate signal yield of this ion counting detector was determined by adjusting the yield value to obtain the accepted ^162^Er/^167^Er ratio (49), when measuring natural Er. Single measurements consisted of 40 repetitions of 15 s long signal integrations at ion beam intensities on mass 167 of ca. 2 pA obtained from ca. 50 ppb (element concentration) solutions of natural Er.

The reported Er isotope ratios of the irradiated Er_2_O_3_ were calculated from the %-deviations of the values obtained on nine bracketing analyses of the natural pre-irradiated Er_2_O_3_ and the accepted isotope composition of natural Er (49) (“bracketing analyses” describe the analyses of natural Er_2_O_3_ that were performed immediately before and after the analyses of the irradiated Er_2_O_3_). Final results represent the average of ten individual measurements of the irradiated Er_2_O_3_. The reported uncertainties are the 95% confidence interval of the reported average values and incorporate the 95% confidence interval of the corresponding average values of the natural Er_2_O_3_ analyses by quadratic addition. Note that any inaccuracy or drift in the yield value of the Daly ion counting detector canceled out, because the irradiated Er_2_O_3_ analyses were evaluated relative to the composition obtained for the bracketing analyses of the natural pre-irradiated Er_2_O_3_. Instrumental background signals were monitored using interspersed analyses of sample-and-standard-free 0.28 M HNO_3_, but required no corrections given the relative nature of the data evaluation and the (mostly) orders of magnitude higher ion beam intensities at the most relevant Er masses.

#### 2.6.3. ICP-OES Measurements

Standard solutions containing Er, Tm, and Zn (0.1, 0.5, 1, 5 ppm) were prepared in 2% HNO_3_ (Merck Suprapur), using Sigma-Aldrich TraceCERT, 1,000 ppm Er, Tm, and Zn ICP standards. Three mass separated samples were characterized using ICP-OES (Agilent ICP-OES 5110) to determine Er, Zn, and Tm concentration of the samples.

## 3. Results and Discussion

### 3.1. Efficiency Assessment With ^169^Tm

**Figure 2:**
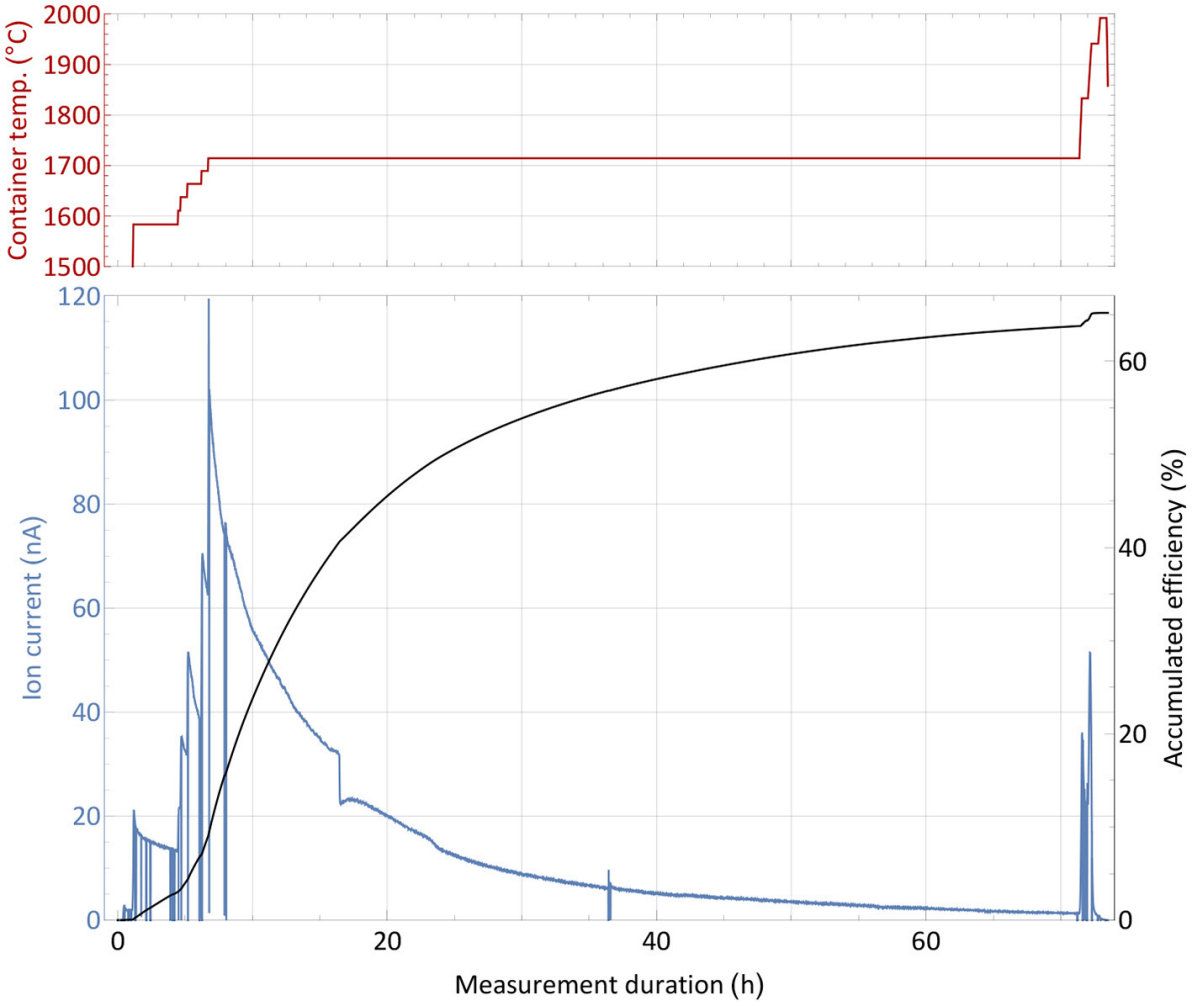
Overall efficiency determination measurement with a 1,590 nAh stable ^169^Tm sample. The extracted ion current is shown with respect to the sample container temperature (top panel) over time. Additionally, the accumulated efficiency (i.e., the ratio of collected atoms to the initial sample amount) is given for every time point.

The efficiency determination measurement with the non-radioactive thulium sample, as described in section 2.3, is depicted in Figure 2. Before the start of the measurement, the ion source was set to a nominal operation temperature of around 2,000°C^2^. The sample container was gradually heated and the ion current on mass 169 monitored. At a low intensity ion beam of a few 10 pA, the operation parameters of the separator and laser system were optimized with direct feedback. Subsequently, the container temperature was increased in steps to investigate the response of the ion signal, which is governed by the supply rate of the atomic fraction into the ion source. Thus, the temperature dependence of the onset of release (around 1,600°C for ion currents of 16 nA, representing collection of 1% of the complete sample per hour) and possible interfering chemical reactions or other effects that would decrease the atomic fraction can be probed. Such effects were not observed. After this first phase of heating, the sample gradually evaporated at a fixed temperature of around 1,700°C, producing a slow exponential decrease in ion current intensity. Through step-wise increase of the temperature, the process of evaporation can be accelerated. Additionally, some parts of the sample which might have condensed at colder spots within the setup were heated out. Once no more significant ion current was detected despite further temperature increase, the sample was treated as depleted and the measurement completed. The chosen overall measurement's duration of 3 days is typical for the collection of radionuclides with a half-life of 5 days or more, such as ^167^Tm. In these collections, the sample heating can be increased gradually over time to allow for less violent outgassing, and decrease total average ion load and stress to the material.

A source of uncertainty in this type of measurement is possible contamination by isobaric ions on the mass of interest, which contribute to the integrated ion current. This would cause an overestimation of the efficiency. Yet, by blocking and unblocking the laser light to the ion source (or changing other laser related parameters as wavelength or pulse timing), sharp, brief drops in ion current can be observed (see Figure 2). The resulting difference in ion current can unambiguously be linked to the element of interest and facilitate the background estimation. During the measurement, this laser enhancement ratio was always >70, i.e., the possible contamination portion was below 2% (and also likely to be surface-ionized thulium itself). Performing a theoretical surface ionization estimation, as described in (50) for a 2,000°C hot rhenium source using an estimated survival parameter of ω = 0.1 as in (51), the rhenium work function Φ = 4.72 eV and the thulium ionization potential of 6.1843 eV, roughly 0.5% thulium surface ionization efficiency from the hot cavity itself could be calculated. In combination with recorded laser enhancement factors at certain points of even >150, this result is consistent with the 65% overall efficiency determined by the complete measurement. While this value presents a reliable assessment, it should not be taken as a precise quantification, as a series of measurements would be required to fully determine its uncertainty. Nevertheless, it enqueues into the series of recently achieved efficiencies on lanthanides as described in the introduction and qualified for the subsequent radiogenic Tm separation tests.

### 3.2. Efficiency Assessment With ^169^Tm in Erbium Oxide

**Figure 3:**
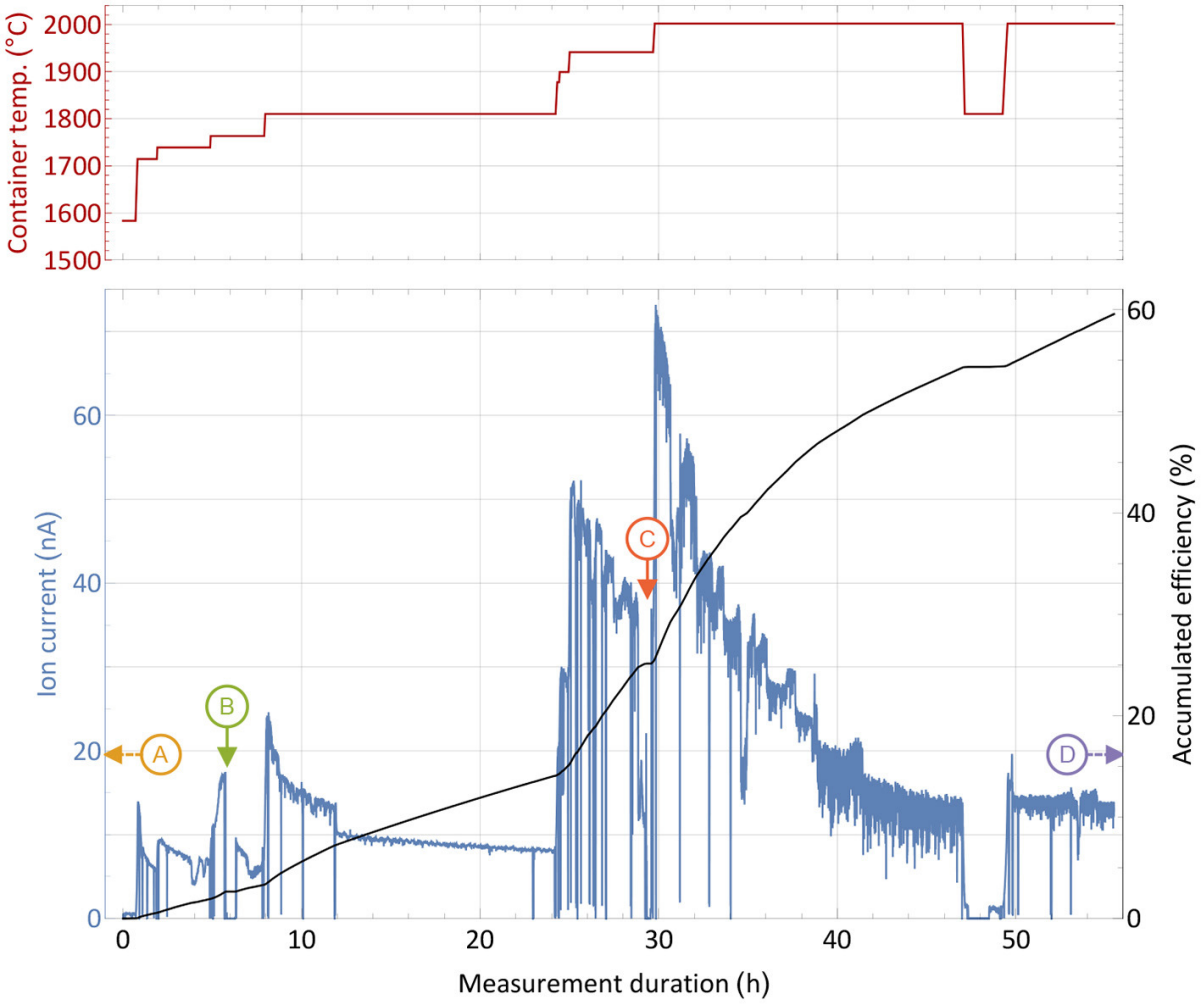
Overall efficiency determination measurement with a 1,590 nAh stable ^169^Tm sample and a 10,000-fold ^nat^Er_2_O_3_ excess in a presentation analog to Figure 2. Instants where the mass scans presented in Figure 4 were taken are marked with capital letters. The extracted ion current is shown with respect to the sample container temperature (top panel) over time.

An efficiency and operation characteristics measurement with the large erbium excess sample was performed in analog way as the one described in section 3.1 and is reported in Figure 3. The sharp and brief drops were caused by either probing the laser enhancement factor or inserting a Faraday cup before the separating magnet to probe the total ion beam current emitted from the source. Additionally, records of the mass spectrum in the erbium and thulium region were performed at different occasions and certain container temperatures.

From the initial period of the measurement, it was apparent that higher temperatures were required for the same extracted ion current compared to the pure thulium sample. A current of around 16 nA, corresponding to 1% thulium extraction per hour, was reached between 1,700 and 1,800°C. In order to raise the extracted current to several 10 nA at later stages, 200–300°C higher temperature had to be applied. Different reasons may explain this difference: The presence of oxygen and of a more oxidative environment may possibly favor thulium oxide molecule formation, and dissociation of these molecules to required atomic species could occur only at higher temperatures. Indeed, equilibrium chemistry calculations using the HSC Chemistry 10 software confirm higher required temperatures for the same amount of thulium gas, yet also on a lower magnitude of around 50°C. The latter can only serve as a rough lead though, as equilibrium conditions are not guaranteed, extrapolation of chemical properties for high temperatures are used and the program is not designed to work reliable in low pressure regimes. In addition, a small deviation can be attributed to the exact sample position, but the overall temperature gradient does not exceed 100°C over the full container length, and a maximum of 30°C for positions near the center, as thermo-electric simulations using ANSYS 2019 R3 show. Finally, the higher ion load due to more surface-ionized erbium in the source can have an influence, leading to a decrease in ionization efficiency of laser-ionized species by providing less confinement in the ion source and thus requiring higher temperature of the setup parts to re-instate the ion survival conditions (50) from the pure thulium sample test. This effect is currently under systematic investigation in view of high throughput laser ion source development.

The most important result is the overall recorded efficiency of 60%, being very close to the value obtained in the pure thulium measurement described in section 2.3. Actually, as seen at the right-hand side of the graph, a considerable thulium ion beam was still present when the machine had to be shut down due to scheduling constraints. An even higher efficiency number for complete depletion of the sample can thus be assumed. This shows that no major interfering effect of the overall evaporation/atomization of thulium by an erbium and oxygen excess exists, besides the shift in temperature which is easy to provide by the setup. Major limitations due to ion load constraints can be neglected as the overall efficiency did not change. The laser enhancement factors varied between 120 in early and 30 in later stages of the measurement.

**Figure 4:**
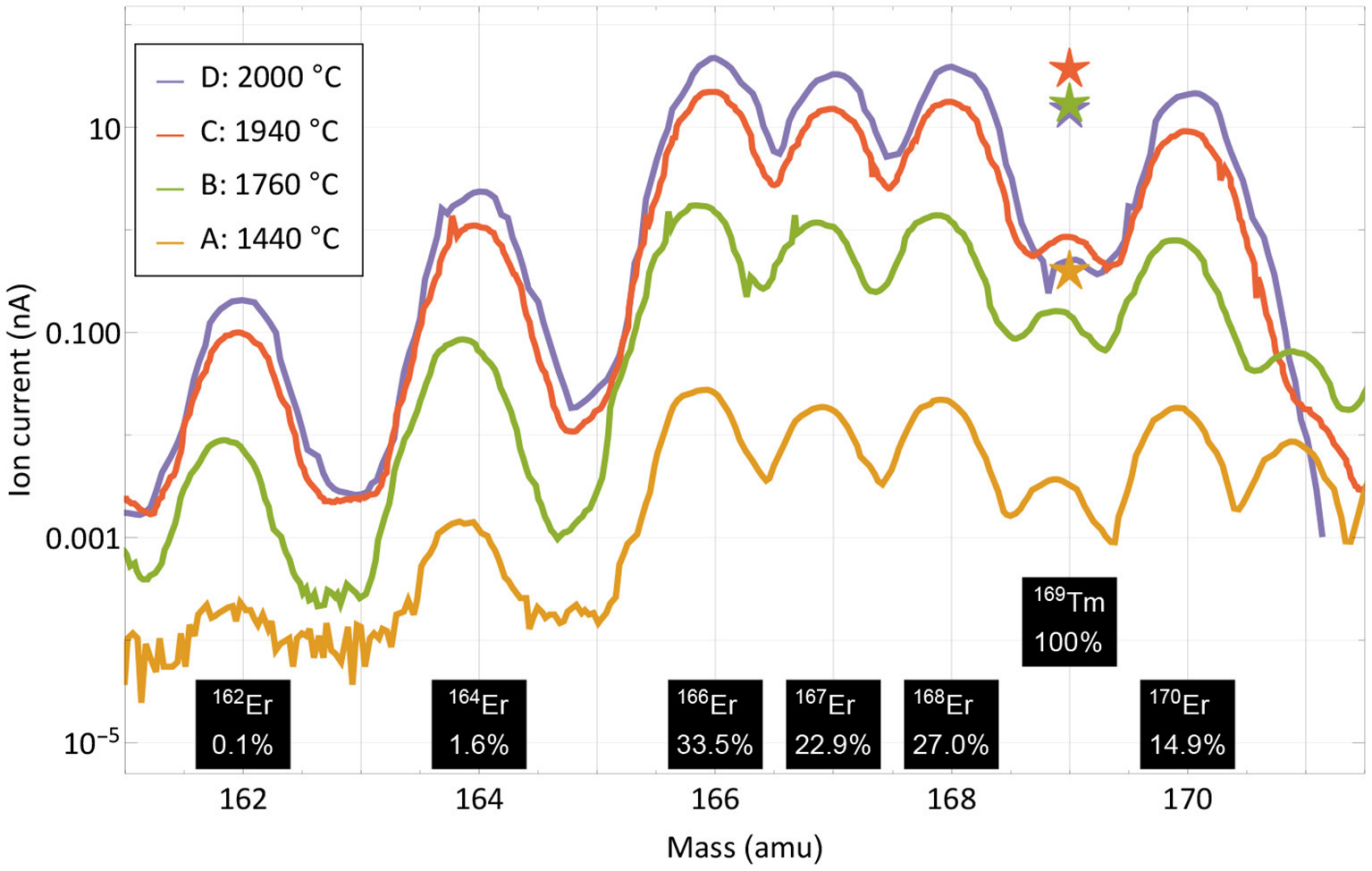
Mass scans of the thulium and erbium region performed at different occasions and temperatures as marked in Figure 3. The scans were performed without lasers, the respective measured ion current on mass 169 with lasers is marked with star markers. Natural abundances of erbium and thulium are indicated.

For the extraction of radioactive ^167^Tm from erbium oxide, the ion beam on mass 167 is dominated by the stable ^167^Er isotope, which has a 22.87% natural abundance. In order to assess a possible purification on this mass by using the element-selective laser ionization, mass scans of the erbium and thulium region were performed at different occasions during the measurement to determine the current ratio in the extracted ion beam. The measurement points were selected at different temperatures and are marked in Figure 3. Mass scan “A” (1,440°C) was performed before the efficiency determination measurement at a point with very low thulium evaporation, mass scan “D” (2,000°C) directly at the end of the measurement. The mass scan data are shown in Figure 4. These scans were performed without lasers, the star markers on mass 169 indicate the respective measured laser ion current at that point.

**Figure 5:**
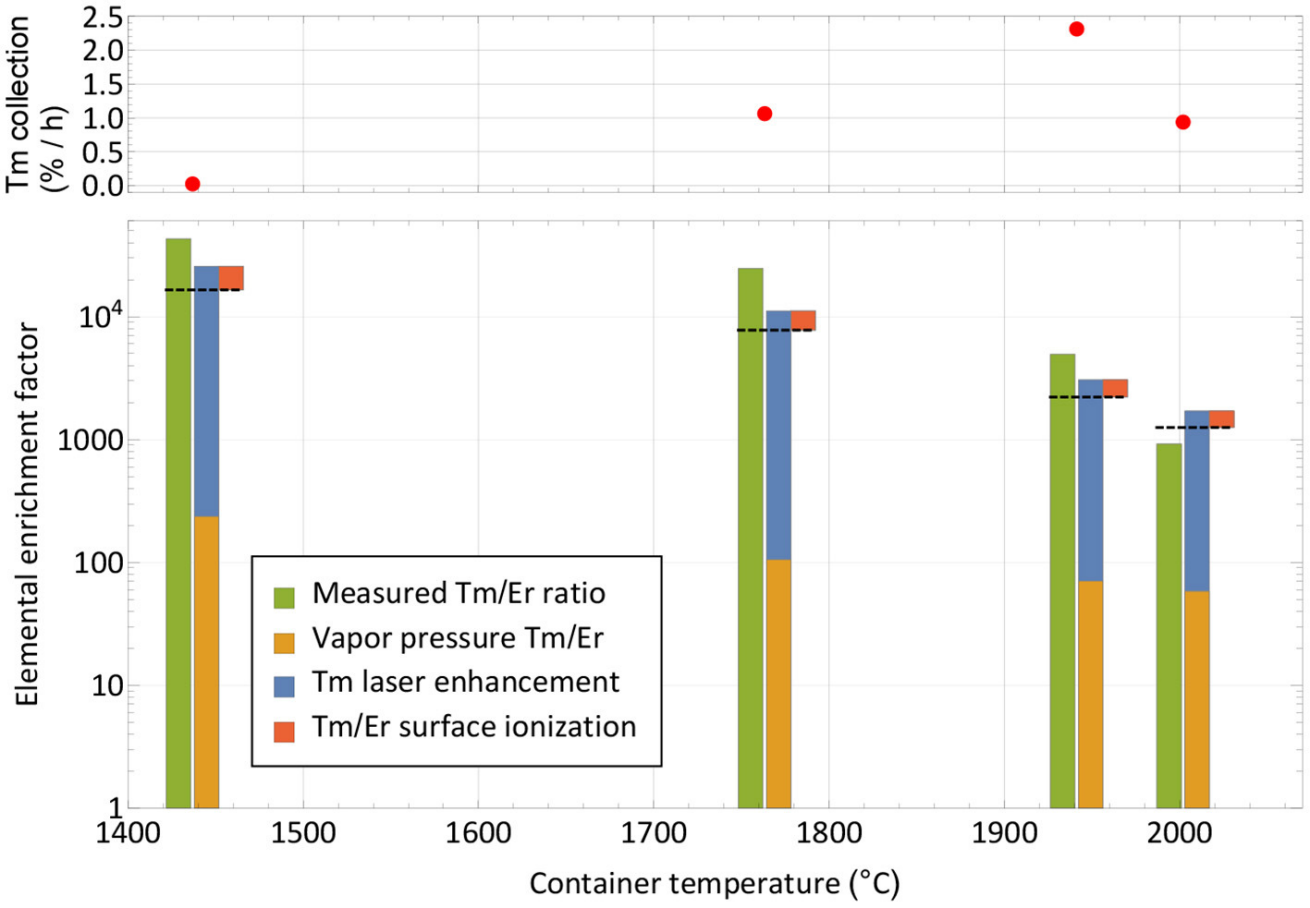
Breakdown of mechanisms involved in the extracted ion beam composition for thulium and erbium. The respective measured elemental enrichment factors (left-hand columns) as extracted from Figure 4 are compared with theoretically expected ones (dashed lines) calculated from vapor pressure, laser enhancement (both favoring thulium) and surface ionization efficiency (favoring erbium). The overall trend is reproduced. The top panel depicts the corresponding collection rate at the points of measurement.

The curves show that the separator output on these masses is governed by surface-ionized erbium with its natural abundance pattern of six stable isotopes in between masses 162 and 170. It is also apparent that both the laser enhancement ratio and the ratio of thulium vs. erbium decrease with higher temperatures. Yet, even without laser enhancement, the relative thulium fraction clearly exceeds the sample composition ratio of 1:8,800; thulium extraction is favored over erbium. A breakdown of the involved mechanisms that lead to respective ion beam extraction of erbium and thulium are presented in Figure 5.

Neglecting deviations from the Knudsen law, the evaporation rate of a sample in atomic form is governed by its vapor pressure, which in turn depends on its temperature (52). The thulium vapor pressure exceeds that of erbium by a factor 240–60 in the investigated temperature range, with a decreasing trend at higher temperatures (53). It is shown as the base of the theoretical elemental purification factor in Figure 5. Furthermore, the element-selective laser resonance ionization only affects thulium. The ratios shown were extracted from the measured ion currents in Figure 4 on mass 169 with and without lasers and reach from 110 for the first two measurements to 40 and 30 for the last two. The decline in enhancement may be attributed to less pronounced laser ion confinement at higher total ion load (50) and enhanced surface ionization of thulium. For the calculation, it is assumed that the mass 169 ion current without lasers is solely surface-ionized thulium. This assumption is justified from the mass peak shapes shown in Figure 4. For example, on the right-hand side of the mass 170 or left-hand side of the mass 166 erbium peaks, it can be seen that, at a neighboring mass, the remaining tailing has a residual intensity reduced by a factor of close to 1,000 compared to the maximum. Transferring this to mass 169 (where erbium has no stable isotope), the admixture of erbium from the two neighboring peak tailings is significantly <10% of its height. Also, no stable isotope of a different element of mass 169 exists. To complete the comparison between thulium and erbium, finally the different surface ionization efficiencies have to be taken into account. Using, again, the model from (50), ionization of erbium is favored by between 60 and 35% for the investigated temperatures. The red top parts in Figure 5 show this factor, which has to be deducted from the previous mechanism all favoring thulium. The expected final overall elemental enrichment factor is then depicted by the dashed horizontal line.

The measured elemental enrichment factors in Figure 5 (left-hand columns) were determined directly by the ratio of the mass 169 thulium ion current (with lasers present) vs. the erbium ion current on mass 167, taking the natural erbium abundances and the sample composition ratio of 1:8,800 into account. These measured values follow the trend of decrease with higher temperature, albeit on an increased level of a factor 2–3 for the first three measurements, and ≈0.7 for the last. For the latter it should be considered that it was performed at the very end of the experiment, where, after already achieving 60% collection efficiency of the thulium sample, it is close to depletion. In contrast, the erbium should have remained at a higher fraction due to the lower evaporation rate. These investigations, together with the achieved efficiency, hint toward that additional effects not covered in the model that would discriminate thulium in an erbium oxide excess environment are not present. In contrary, molecule formation e.g., on oxide sidebands leading to losses in the atomic form, or permanent adsorption onto the container or ion source material, seem to affect erbium more severely. It should be noted though that on the mono-oxide sideband of erbium the extracted ion current was a factor >100 smaller than on the atomic fraction (but the ionization efficiency ratio is unknown), and the calculated surface ionization efficiency for erbium is also consistent with results obtained in (54) for the respective setup. Thus, part of the discrepancy may also be attributed to the rather crude model approach and potential partial inappropriateness in the vapor pressure data for the actual experimental environment.

The resulting measured elemental enrichment factors between 1,000 and a few 10,000s show the capability of using a mass separator in combination with element-selective (laser resonance) ionization not only for boosting the efficiency and purity of the product itself by mass separation, but also the performance under isobaric contamination. In the presented case, thulium evaporation was favored at lower temperatures, enabling us to perform the collections before the main part of the contaminant is extracted. Should the opposite be the case, a large fraction of the contaminant could be extracted and dumped at low temperatures, while preserving the sample of interest. The limits for both treatments (and especially the latter) are time constraints for the extraction of the sample. This is also depicted in the top panel of Figure 5, where the thulium extraction rate at this point in time is given. Increasing the temperature is mandatory for keeping a reasonable extraction rate over time and achieving full release and depletion of the isotope of interest.

### 3.3. Efficiency Assessment With ^167^Tm From Proton-Irradiated Erbium Oxide

**Figure 6:**
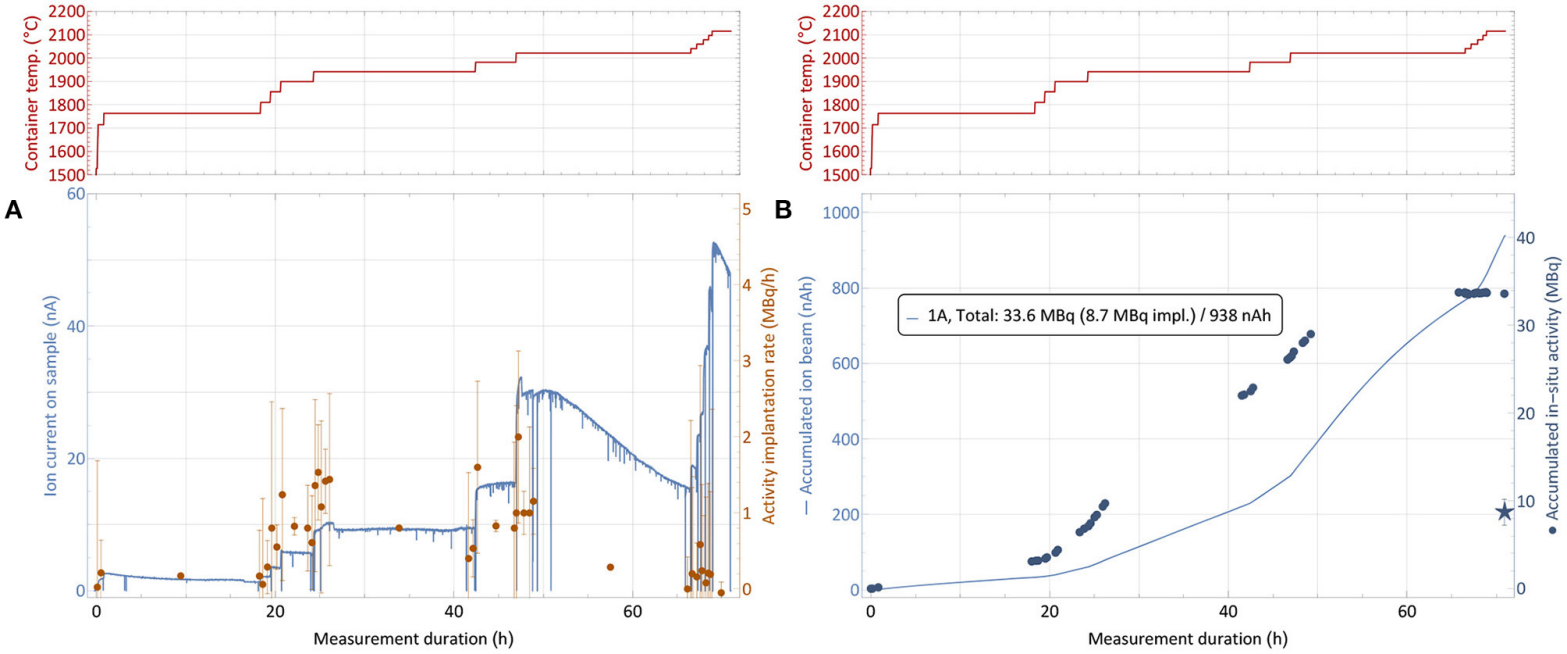
^167^Tm collection process for sample 1 (82.5 MBq ^167^Tm, see Table 1). The top panels show the sample container temperature over time. The bottom left graph **(A)** depicts the ion current measured on the implantation foil and the rate of ^167^Tm activity implantation. The bottom right graph **(B)** depicts the respective accumulated values. Due to sputtering effects of the high intensity ion beam, only a fraction of the activity measured to be present in the collection chamber remained on the foils, as shown by the star marker that depicts the gamma-ray spectroscopy measurements of the foils after collection. Errors on the activity are 95% confidence intervals of constant 0.2 MBq for the activity which are propagated into the rates. The inset in **(B)** describes the measured total *in-situ* activity in comparison to the gamma-ray spectrometry of the foil afterwards and the integrated intake of ion beam.

The collection process of sample number 1, as described in section 2.5, is shown in Figure 6. The top panels show the step-wise heating of the sample container to successively release the thulium into the ion source. The left-hand bottom graph depicts the corresponding evolution of both the ion current measured on the implantation foil and the rate of ^167^Tm activity present on the foil. The latter is derived from *in-situ* gamma-spectroscopic activity determination at certain points in time, which are shown on the right-hand bottom graph. It is complemented by the integrated ion current as measure of overall deposited atoms. Up to three different implantation foils were used per run, one foil each only receiving low activity, which was afterwards used for internal radiochemistry tests and not shipped back to PSI.

Starting from a container temperature of around 1,700°C as derived as onset of significant thulium extraction in the separation development (section 3.2), the temperature was increased further while the amount of activity was measured in regular intervals. Respective activity implantation rates were calculated as shown in Figure 6A, with the errors propagated from the 95% confidence intervals of 0.2 MBq on the total activity throughout the measurements given by the detector. The point of 1% ^167^Tm extraction rate, which was measured at 1,700°C with stable ^167^Tm in ^nat^Er_2_O_3_ environment, was observed to be shifted to roughly 1,800–1,900°C. These higher required temperature can be caused by the fact that this time the thulium had to diffuse out of the erbium pellet first. Additionally, the even higher excess of erbium and oxygen might shift the effective dissociation point of oxide molecules.

After reaching the onset point of thulium extraction, the container temperature was gradually increased further to evaporate all thulium within the scheduled separation time. As already described in section 3.2, it can be seen that the ratio of thulium in the extracted mass 167 ion beam (which predominantly consists of surface-ionized stable ^167^Er) decreases over time, with sample depletion and with increasing temperature. For ^167^Tm, a collection rate of 1 MBq/h corresponds to an ion beam current of around 50 pA. Thus, in Figure 6A, an identical y-axis height of ion current and activity rate numbers corresponds to a ≈ 1:250 ratio of ^167^Tm:^167^Er. Taking the 23% natural abundance of ^167^Er into account, this ratio corresponds to an overall ^167^Tm:^nat^Er purification factor of around 1,000. Throughout the three collections, actual purification rates between a factor 2 higher (low temperatures) and a factor 10 lower (high temperatures, exhausting thulium sample) than this value were recorded. As expected, these results follow the same trend as in Figure 5, showing no difference in behavior between the test and actual collections. The overall reduction of a factor 10 in comparison with the elemental enrichment rates in the preparatory, non-radioactive test can most probably be attributed to the above mentioned effect of a large fraction of thulium being enclosed inside the erbium pellet, leading to reduced evaporation rates.

The ^167^Tm separation efficiency as a crucial performance parameter determined by comparing the measured activity on the collection foil to the initial activity in the irradiated sample at the start of the collection. Figure 6B shows the activity at the implantation point for each of the foils from *in-situ* measurements, determined by the difference between the readout at a given point in time and the background value at the time when this foil was moved into the beam. In previous MEDICIS runs with different elements (from a few up to more than 100 MBq of ^153^Sm, ^155^Tb, ^225^Ac) on identical collection foils (34), this value proved to be a reliable estimate for the gamma-spectrometry measurements that are performed on the foils afterwards by independent measurements in a dedicated, external setup. Yet, for the presented case this value obtained after recuperation of the foil, shown as star marker in Figure 6B with a 95% confidence interval error band, was significantly lower. Only 8.6 (15) MBq implanted in the main foil were measured, in contrast to 33.6 MBq as estimated from the *in-situ* determinations. Loss of the main fraction onto the collimator by a fault in the ion optics was excluded by the ratio of respectively measured ion currents, with <3% of the overall current recorded on the collimator. An overview of the respective activities and efficiencies for all runs is given in Table 1.

**Table 1:** Overview of the three ^167^Tm collections performed at MEDICIS.

			**Separated ^167^Tm**			**Collected ^167^Tm**			
**No**.	**^167^Tm content^a^**	**Foil**	**Activity per foil^b^**	**Total run activity^c^**	**Dec.-corr.^d^ (%)**	**Activity per foil^b^**	**Total run activity^c^**	**Dec.-corr.^d^ (%)**	**Ion load^e^**
	**(MBq)**		**(MBq)**	**(MBq)**		**(MBq)**	**(MBq)**		**(nAh)**
1	83.0 (80)	1A	33.6	33.8 → 41%	51	8.7	**8.9(15) → 11(2)%**	13	938
		1B	0.2			0.2			1
2	76.9 (73)	2A	14.6	34.4 → 45%	55	7.5	**15.4(19) → 20(3)%**	24	208
		2B	20.2			7.7			919
		2C	0.5			0.7			5
3	122.9 (118)	3A	28.8	33.1 → 27%	32	15.4	**19.2(25) → 16(3)%**	19	496
		3B	2.9			2.9			45
		3C	4.0			2.4			504

*The mass 167 ion beam of three proton-irradiated erbium oxide pellets was implanted into two or three foils each per run. The activity on the foils was determined by in-situ gamma-spectroscopy in the collection chamber (“Separated ^167^Tm”) and dedicated gamma-ray spectroscopy measurements on the foils after retrieval (“Collected ^167^Tm”). Respective efficiencies and uncertainties where applicable are given and discussed in the text*.

a *At start of collection run*.

b *At end of individual foil implantation*.

c *Sum of foil activities at end of implantation of the last foil (end of run). Efficiency given as ratio to sample activity at start of run*.

d *Separation efficiency corrected for radioactive decay*.

e *Cumulated ion beam intake*.

*The reason for bold typesetting is to mark these numbers as main result*.

The “total run efficiency” describes the ratio of the activity available at the overall end of the run compared to the available activity at its start. If single foils would be extracted directly at the end of their individual implantation (with the “activity per foil” at that time,) instead of all together at the end of the run, a small benefit in avoiding decay losses while implanting the other foils would be gained; thus their sum is higher than the total efficiency. Additionally, the “decay-corrected efficiency” as technical separator performance parameter is given by calculating the theoretical efficiency if no decay was present.

**Figure 7:**
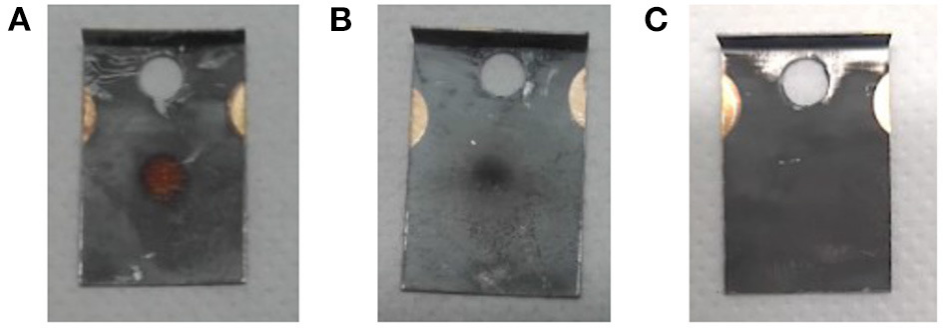
Effect of high ion beam load on the implantation foils. **(A)** Foil 2B: 919 nAh, **(B)** foil 2A: 208 nAh, **(C)** foil from different radionuclide implantation with <30 nAh ion beam load as comparison.

Visual inspection of the foil (Figure 7) and comparison to operation beam parameters (especially ion beam intensity) of previous MEDICIS runs identified sputtering effects on the foil as dominant loss factor. Due to the high ion beam intensity separated on the same mass of collection, some fraction of already implanted ^167^Tm was released from the foil and condensed onto the structures in the collection chamber. The *in-situ* gamma-activity measurement was able to detect this fraction (albeit with an unknown, lower efficiency due to a different geometrical distribution further away from the implantation point for which the detector was calibrated—for this reason also error bars on the *in-situ* activities can not be quantified), which could not be recuperated with the foils any longer: a measurement on the empty foil holder frame after the first implantation yielded 20.8 (36) MBq, and later measurements with the *in-situ* detector of the empty collection chamber yielded an apparent value between 6 and 7 MBq. These results show that the *in-situ* detector provides a reliable value for the extracted ^167^Tm activity within the collection chamber and can be used to determine an “extraction and implantation point delivery” efficiency, in contrast to the actual implanted activity remaining on the foils.

**Figure 8:**
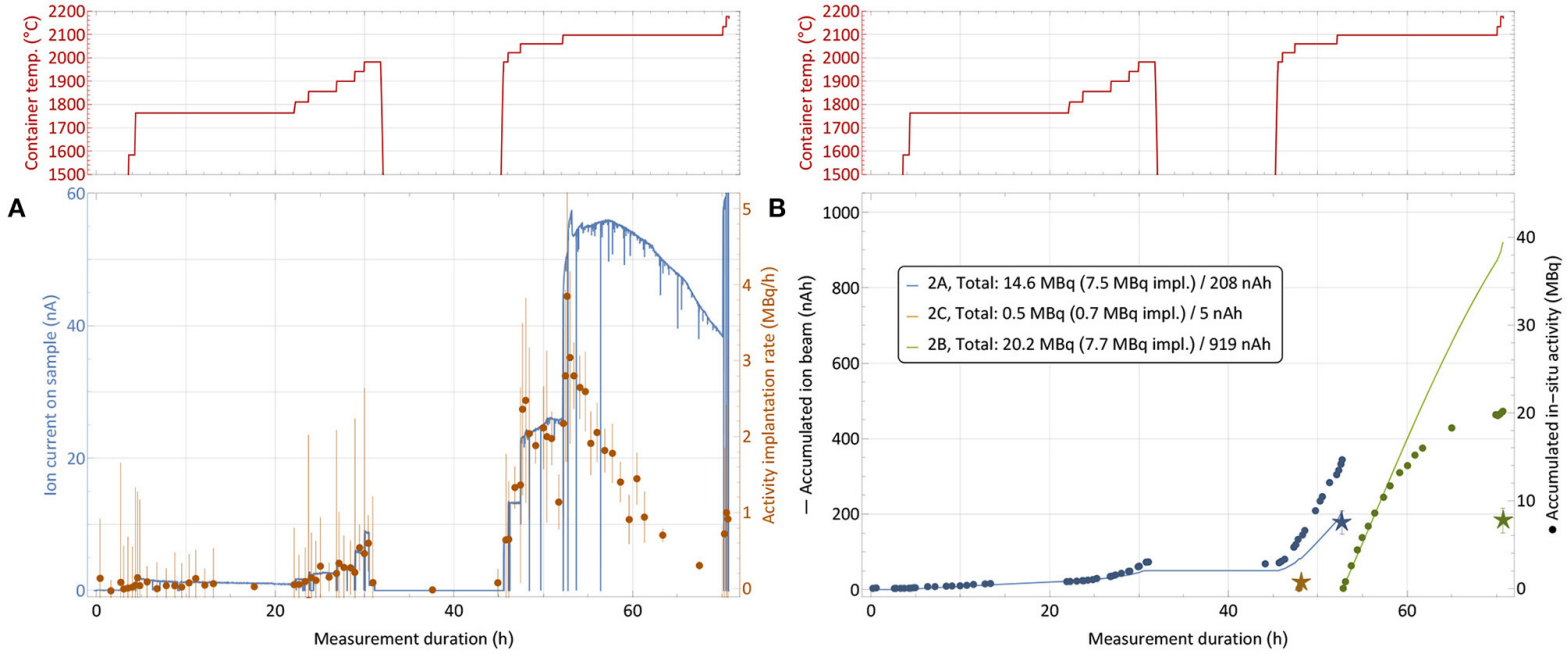
^167^Tm collection process for sample 2 (76.9 MBq ^167^Tm, see Table 1) with distribution over multiple implantation foils. Depiction analogous to Figure 6. The break between 32 and 45 h with cooled down container is due to a technical intervention.

In order to mitigate this effect with *ad-hoc* methods in the immediate two subsequent collections, the implantation process was distributed over two foils (in addition to the low-activity one for internal use). The implantation timeline on sample 2 is depicted in Figure 8. While at the end of the first run only around 25% of the *in-situ* measured activity was found on the implantation foil, in this configuration fractions of close to 50% (foil 2A) and 40% (foil 2B) were achieved. The low-activity implantation foil 2C, which received very limited integrated ion beam exposure, did not show any loss between the *in-situ* and a dedicated subsequent measurement.

Table 1, where *in-situ* and actual implanted activities are compared, also gives the overall integrated charge measured on the implantation foils as an easy-to-access metric of ion beam exposure. A complete description would require the time evolution of the ion beam intensity as well. The correlation between higher fractions of activity remaining on the foil and low integrated charge can clearly be established. For run 3, the ion beam load was distributed as equally as possible on the two available implantation foils (foil 3C was for internal low-activity use), and additionally the beam position was slightly moved at various occasions to avoid exposure of the same spot to the intense beam center for the whole duration. With these measures, in run 2 and 3, 45% (15.5 out of 34.4 MBq measured *in-situ*) and 58% (19.2/33.1) of the extracted activity remained on the foil, compared to only 26% (8.9/33.6) for the first run. Follow-up comparison studies using aluminum and copper layers on the collections foils instead of zinc preliminarily yielded in between 75 and 80% of *in-situ* measured activity remaining on the foil after retrieval for aluminum, while in the case of copper only a minor amount of around 10% was determined for similar ion current exposures.

Taking the extracted ^167^Tm as performance of the process without implantation into account, these results prove that an extraction and implantation point delivery efficiency of ^167^Tm from an irradiated ^nat^Er_2_O_3_ target between 27 and 45% is possible at MEDICIS if sputtering can be avoided. The decay-corrected efficiency can be compared to the tests with stable ^169^Tm (60% efficiency), and achieved values of above 50% show that no strong additional effects occur in the case of irradiated ^nat^Er_2_O_3_.

The actual achieved collection efficiency values of 11%, which could be increased to 20% in subsequent collections by *ad-hoc* implementation of sputtering mitigation procedures, already exceed the 5% goal value on lanthanides for the first stage of MEDICIS and even meet the 20% in scope of a facility upgrade (32). The influence and mitigation of sputtering effects induced by high intensity beams are currently subject to dedicated developments, including different implantation materials as mentioned above, automated permanent beam movement to avoid a focus on the same spot for the whole duration, and chemical purification of the samples beforehand to remove isobaric contaminants in the matrix.

### 3.4. Sample Characterization

Different techniques were applied on both the initial ^nat^Er_2_O_3_ targets and the mass-separated samples at MEDICIS and PSI to characterize and quantify the production and separation process with a number of criteria. An overview of the type of measurements performed on specific samples is given in Table 2.

**Table 2:** Overview of the characterization for different samples.

**Samples**	**Implanted foils**	**Characterization of the samples**			
		**Gamma-ray** **spectrometry** **(^167^Tm activity)**	**ICP-MS** **(Isotope ratio)**	**ICP-OES** **(Er-concentration)**	**Gamma-ray** **spectrometry** **(^168^Tm activity)**
^nat^Er_2_O_3_			x		
Test target		x	x		
Target 1	1A	x	x	x	
	1B	x			
Target 2	2A	x			x
	2B	x	x	x	
	2C	x			
Target 3	3A	x	x	x	
	3B	x			
	3C	x			x

#### 3.4.1. Activity Measurements

**Figure 9:**
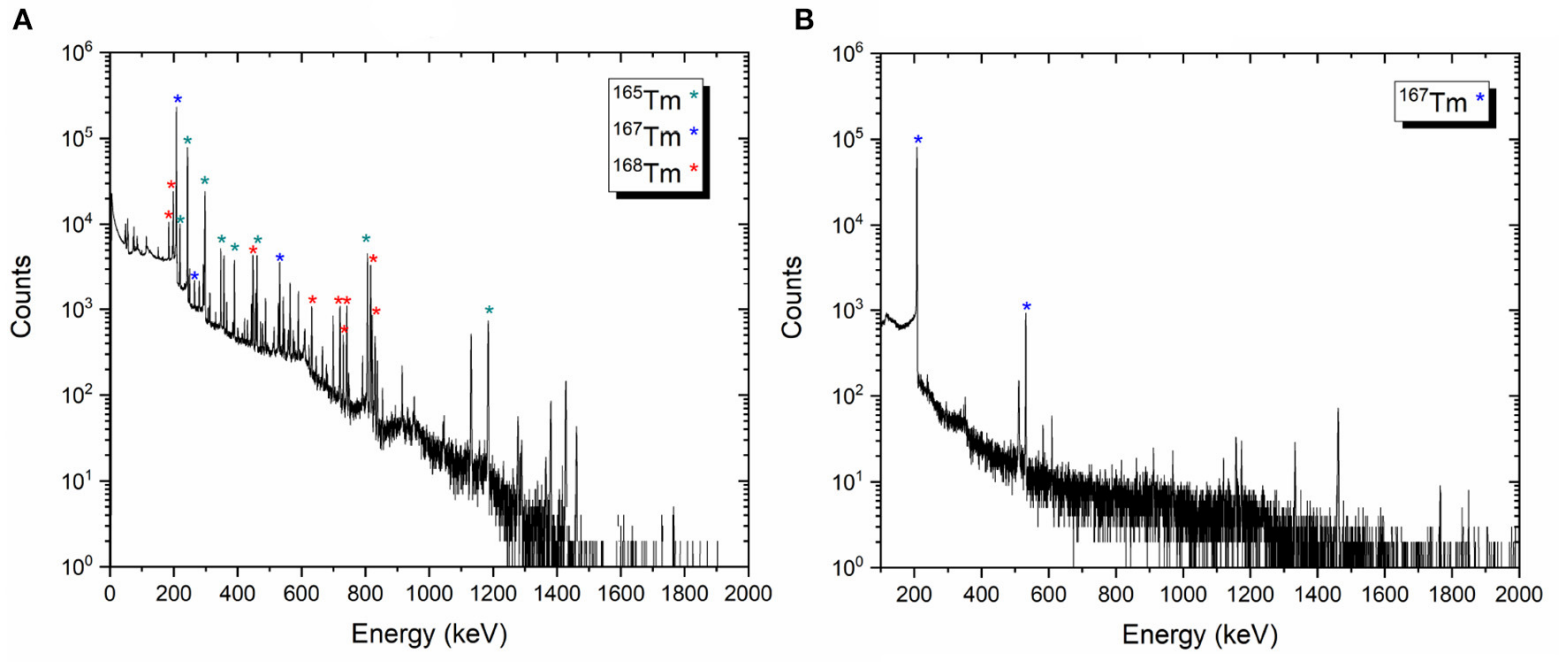
Examples of the gamma-ray spectra of the target material before mass separation process on target 2 **(A)** and ^167^Tm implanted foil 2A **(B)**.

Representative gamma-ray spectra of the samples before and after mass separation, performed at PSI, are shown in Figure 9. At the time of the measurement, no ^166^Tm activity was determined due to the 7 days cooling time (Figure 9A).

The activities of the other thulium radionuclides measured for each target were given as follows:

- Target 1 [^167^Tm: 106.13 (1,021) MBq, ^168^Tm: 4.50 (19) MBq, ^165^Tm: 41.04 (216) MBq]
- Target 2 [^167^Tm: 97.81 (929) MBq, ^168^Tm: 8.74 (29) MBq, ^165^Tm: 46.26 (243) MBq]
- Target 3 [^167^Tm: 154.37 (1,482) MBq, ^168^Tm: 8.67 (26) MBq, ^165^Tm: 61.92 (325) MBq]

**Figure 10:**
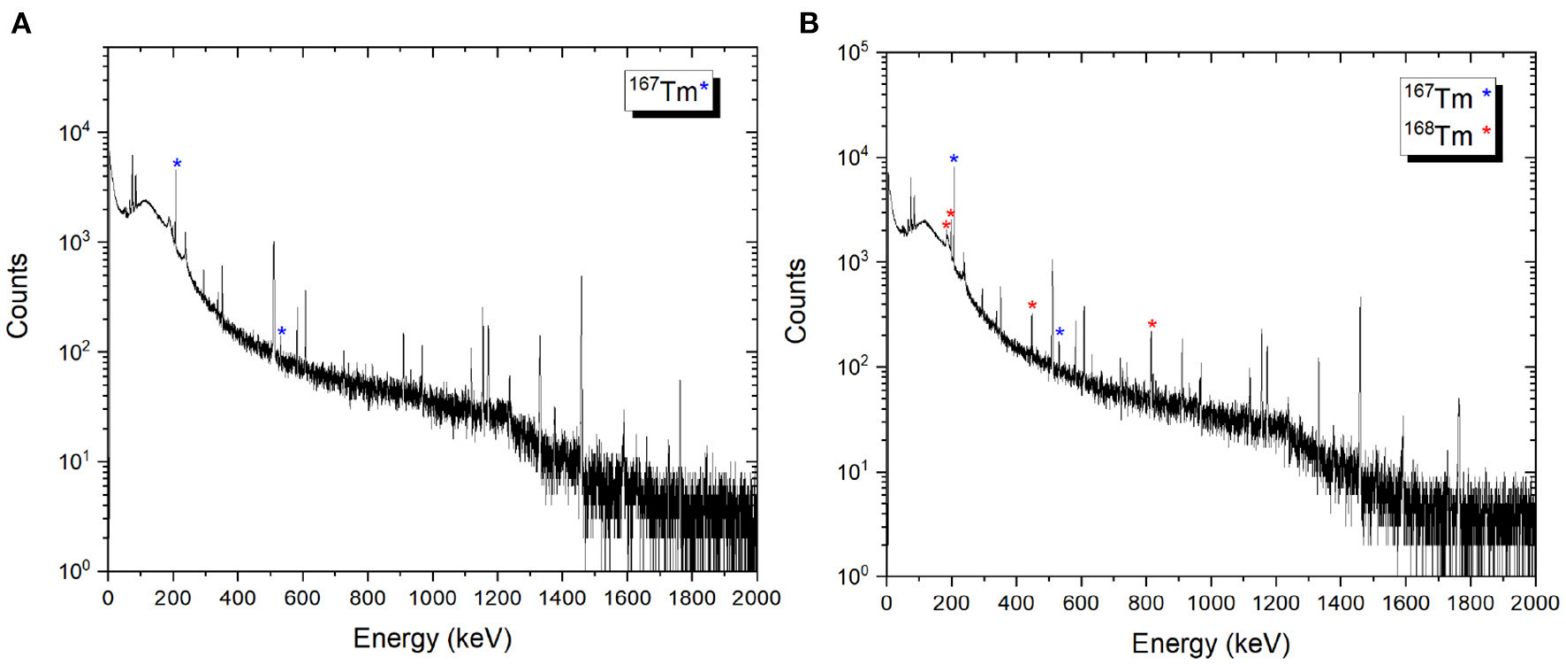
Examples of gamma spectra of the ^167^Tm implanted foils 2A **(A)** and 3C **(B)**. The measurements were performed 3 months after mass separation, using the same sample detector distance and counting time.

After the mass separation process, the ^168^Tm activity of the samples could not be determined due to the high ^167^Tm activity (Figure 9B). Therefore, gamma-ray spectrometry measurements of Sample 2A and 3C were repeated 3 months after the mass separation process (Figure 10). No ^168^Tm peak was visible for Sample 2A, while 912 Bq ^168^Tm was detected in Sample 3C. This activity corresponds to 0.04% radionuclidic impurity after the mass separation process, and respectively a suppression factor of around 200.

After dissolving the zinc layer of samples 1A, 2B, and 3A, the gold foils were re-measured using gamma-ray spectrometry to determine the remaining ^167^Tm activity on the foils themselves. The results showed <2% of the total ^167^Tm activity at the time of the first gamma-ray spectrometry measurement.

#### 3.4.2. Isotope Ratio Measurements

In order to quantify the mass separation power of the process, the relative abundances of erbium isotopes were compared before and after mass separation, i.e., in the proton-irradiated test target and in the foils.

##### 3.4.2.1. Natural Er_2_O_3_ and Proton-Irradiated Er_2_O_3_ Samples

The composition of natural and proton-irradiated Er_2_O_3_ obtained using the mass discrimination correction described in section 2.6.2 are identical within 95% confidence. Therefore, additional average values using a more precise entirely internal mass discrimination correction based on the measured ^170^Er/^166^Er are also reported for information. An overview is given in Table 3. Note that this correction scheme must fail, introducing artificial deviations from natural isotope ratios, if the ^170^Er/^166^Er of the irradiated Er_2_O_3_ was altered by irradiation. Using this more precise correction scheme and with the possible exception of ^164^Er/^167^Er, all isotope ratios of natural and irradiated Er_2_O_3_ are also identical within the given uncertainties.

**Table 3:** Comparison of the isotope ratios obtained by ICP-MS for natural and proton-irradiated Er_2_O_3_ samples.

	**^170^Er/^167^Er**	**^168^Er/^167^Er**	**^166^Er/^167^Er**	**^164^Er/^167^Er**	**^162^Er/^167^Er**
^nat^Er_2_O_3_^†^	0.65197 (25)	1.17968 (14)	1.46500 (26)	0.070007 (21)	0.006078 (31)
Irr. ^nat^Er_2_O_3_^*†^	0.65200 (30)	1.17969 (17)	1.46503 (27)	0.069994 (26)	0.006074 (32)
Irr. ^nat^Er_2_O_3_^**†^	0.65200 (25)	1.17969 (15)	1.46503 (27)	0.069994 (22)	0.006074 (32)

*Errors given as 95% confidence interval (CI)*.

† *From (49), 95% CI recalculated using N = 20*.

*† *Using relation of mass discrimination factors for Er and admixed Lu from analyses of natural Er_2_O_3_ analyses and including uncertainties from (49)*.

**† *Using measured mass discrimination factor from^170^Er/^166^Er and including uncertainties from (49)*.

##### 3.4.2.2. Mass-Separated Samples

An overview of the isotopic ratios in the implanted foils after mass separation in comparison to the ones in the targets as described above is given in Table 4. For the foils 1A and 3A, the neighboring mass suppression at mass 167 is more than a factor 1,000, with the exception of ^168^Er suppression being around 600. These values agree well with the mass peak tailings of the MEDICIS mass separator investigated in section 3.2, Figure 4. For foil 2B, the respective suppression factors are only between 200 and 300. Post-analyses of the recorded operation parameters of the separator for this run showed no significant change in ion beam shape. A possible explanation is the high ion beam intensity for this implantation at the end of the run whereas the other foils were implanted at the respective beginning, with lower intensity. Sputtering effects of the foil are, thus, more pronounced, especially at the center of the Gaussian shaped beam. Already implanted ^167^Er at this spot is removed more strongly than neighboring mass ions tailing in at the sides. As this possible bias induced by position-dependant sputtering in the foil should also be present in the 1A and 3A foils, albeit less pronounced due to lower ion beam intensity, the extracted neighboring mass suppression factors only present a lower limit to the actual separator performance. The gamma-ray spectrometry results on the ^168^Tm/^167^Tm ratio in foil 3C, also being implanted at a late stage with high ion beam intensity, with a suppression factor of around 200, are in line with these results.

**Table 4:** Comparison of the ICP-MS results of the natural and mass 167 separated samples (1A, 2B, and 3A).

**Samples**	**^**166**^Er/^**167**^Er**	**^**168**^Er/^**167**^Er**	**^**170**^Er/^**167**^Er**
^nat^Er_2_O_3_	1.4650 (3)	1.1797(1)	0.6520 (2)
1A	0.00097 (4)	0.00105 (3)	0.00034 (2)
2B	0.0052 (2)	0.0060 (1)	0.0022 (1)
3A	0.00141 (8)	0.00202 (6)	0.00058 (3)

*Errors given as 95% confidence interval*.

The presence of ^170^Er, with comparable suppression factors as the direct neighboring masses and also a reduced suppression for foil 2B, is unexpected. The mass scans in Figure 4 show no hints of such a tailing over three mass units. Contamination of the foil by other means before the measurements or presence of an unidentified species at mass 170 have to be considered.

#### 3.4.3. Erbium Content of the Mass-Separated Samples

After complete decay of ^167^Tm, the Er and Zn concentration of the samples 1A, 2B, and 3A were determined using ICP-OES. The results are shown in Table 5 together with the calculated ^167^Tm amount at time after mass separation.

**Table 5:** Erbium and zinc remaining traces in the mass separated samples.

	**Implanted Tm (measured)**					**Extracted Tm (calculated)**					**Target**
**Foil**	**Zn**	**Er**	**^**167**^Tm**	**^**167**^Tm**	**^**167**^Tm/Er**	**Er**	**Er**	**^**167**^Tm**	**^**167**^Tm**	**^**167**^Tm/Er**	**^**167**^Tm/Er**
	**(μg)**	**(μg)**	**(MBq)**	**(ng)**	**(MBq/μg)**	**(nAh)**	**(μg)**	**(MBq)**	**(ng)**	**(MBq/μg)**	**(MBq/μg)**
1A	0.4	1.9	8.7	2.8	4.6	938	5.8	33.6	10.7	4.6	3.1 × 10^−3^
2B	0.2	0.8	7.7	2.5	9.6	919	5.7	20.2	6.5	3.4	2.8 × 10^−3^
3A	0.5	1.4	15.4	4.9	11.0	496	3.1	28.8	9.2	9.4	4.7 × 10^−3^

^167^*Tm/Er ratios are calculated for the time directly after mass separation and compared to expected results on extracted activity if sputtering is avoided, and to the ratio in the Er_2_O_3_ targets before mass separation*.

Isobaric ^167^Er cannot be mass-separated and is present with a few 100-fold higher quantity. The results are compared with the expected values based on the extracted ^167^Tm and cumulative ion load (^167^Er) as described in section 3.3, yielding similar values for foils 1A and 3A. Foil 2B shows some differences between measured and calculated values, although being implanted with the same amount of ions as foil 1A. As discussed above, it was implanted at the end of the run with higher ion beam intensity, apparently causing a stronger loss of Er than Tm under these conditions. Yet, for all foils, the separation increases the ^167^Tm/Er ratio compared to the value in the irradiated Er_2_O_3_ targets by a factor of at least 1,500, being consistent with the enrichment factors obtained as described in sections 3.2 and 3.3. Taking only mass 167 into account (i.e., no mass separation but purification due to element-selective laser ionization and vapor pressure differences), the factor is between 300 and 800 for the investigated foils.

The results obtained from the implanted foils show that additional Er removal after the mass separation process is required to use this solution for radiolabeling of different compounds (in MBq/nmol range) to investigate the ^167^Tm potential with preclinical studies. In addition, ICP-OES results showed that the DGA resin was not sufficient for complete removal of Zn.

## 4. Summary and Outlook

This work provides a detailed description on the steps followed to establish the mass separation process of external ^167^Tm samples, produced by proton irradiation of erbium oxide at PSI, at the CERN-MEDICIS facility. Initial tests with non-radioactive thulium samples allowed for optimization of operation parameters and procedures. Separation efficiencies of 65% for a pure thulium sample and 60% for a sample with 10,000-fold erbium excess in these tests proved the adequacy of the setup. The favorable release and ionization of thulium over erbium by utilizing laser resonance ionization and differences in vapor pressures was quantified to reach the regime of 10^3^–10^4^ enrichment factors, and major additional limitations such as undesired molecule formation could be excluded.

Three mass separations to extract ^167^Tm from proton-irradiated erbium oxide pellets in the order of 100 MBq each yielded collection efficiencies between 11 and 20%. The limiting factor proved to be sputtering of the collected fraction induced by the high intensity erbium fraction in the mass separated ion beam, which was for the first time observed in this extent at MEDICIS. *Ad-hoc* mitigation procedures were implemented already after the first run. *In-situ* monitoring of the activity in the collection chamber, thus including the majority of the sputtering losses which were deposited in the direct vicinity, showed that collection efficiencies in the order of 45% are possible if sputtering can be avoided. Mitigation methods as different implantation materials and automated ion beam movement are under investigation and expected to improve MEDICIS' performance in general. An initial test of aluminum instead of zinc yielded a decrease of sputtering loss to around 20%. Further tests are planned to be performed using graphite as implantation material. The decay-deconvoluted separation efficiencies with radiogenic ^167^Tm samples of up 55% show that no strong additional effects as molecule formation or diffusion phenomena in the target matrix occur in comparison with the cold tests, even though the erbium fraction is even higher. Both the actually implanted 11–20% as well as the projected 45% extraction efficiency are to date the highest achieved values with external sources at the MEDICIS facility.

Comparative analyses of the targets and samples before and after mass separation show neighboring mass suppression factors of more than 1,000. Lower values also occur, which might be traced back to sputtering effects as well. The ^167^Tm/Er ratio of the samples could be improved by more than a factor of 1,000 by combining mass separation, differences in vapor pressure and selective laser ionization. Yet, isobaric ^167^Er is still present in a few 100-fold excess and needs to be chemically separated afterwards for application in targeted radionuclide therapy.

The project of providing high specific ^167^Tm activity for medical research will continue by evaluating alternative production paths. Isotopically enriched erbium oxide, the ^nat^Yb(p,xn)^167^Lu → ^167^Tm production route, and spallation of a tantalum target, with respective mass separation process efficiency and sample quality, will be investigated at PSI and at MEDICIS in the near future. The development of accessible and efficient ^167^Tm production routes will allow the use of this radionuclide for preclinical studies in combination with already developed ligands aimed to be used for the targeting of tumors. Preclinical comparison studies of ^167^Tm with other Auger electron emitting radiolanthanides such as ^165^Er and ^155^Tb will have a high impact on the understanding of the therapeutic effects of Auger and conversion electrons.

## Data Availability Statement

The raw data supporting the conclusions of this article will be made available by the authors, without undue reservation.

## Author Contributions

ZT designed the study. ZT, CD, RH, and TS coordinated the study. EC and LL operated the mass separator. RH, KC, and SW operated the laser ionization system. PS performed the ICP-MS analyses. MT assisted the radiochemistry experiments. HZ optimized the proton beam parameter. SH performed target unit temperature simulations. BL performed ion source chemistry calculations. RH and ZT analyzed the data and drafted the manuscript. TC, VF, BM, NM, and TS provided supervision. All authors discussed and reviewed the manuscript.

## Funding

This research received funding from the Swiss National Science Foundation (SNF Grant Number: 200021_188495), the Research Foundation Flanders FWO (Belgium) under contracts FWO SBO Tb-IRMA-V No. S005019N and WO IRI ISOLDE No. I002619N, and the European Union's Horizon 2020 research and innovation programme under grant agreement No. 101008571 (PRISMAP - The European medical radionuclides programme).

## Conflict of Interest

The authors declare that the research was conducted in the absence of any commercial or financial relationships that could be construed as a potential conflict of interest.

## Publisher's Note

All claims expressed in this article are solely those of the authors and do not necessarily represent those of their affiliated organizations, or those of the publisher, the editors and the reviewers. Any product that may be evaluated in this article, or claim that may be made by its manufacturer, is not guaranteed or endorsed by the publisher.

## Footnotes

^1^Mostamand M, Li R, Romans J, Lassen J. Odd-parity Rydberg and autoionizing states of thulium studies by laser resonance ionization spectroscopy. *Spectrochim Acta B*. (Submitted).

^2^Note that this and all further temperature specifications are inferred from optical pyrometer measurements performed after construction of the container/ion source unit and their calibration to the applied current for ohmic heating. For temperatures above 2,000°C, extrapolation of this calibration is used.
